# A Standardized Critical Size Defect Model in Normal and Osteoporotic Rats to Evaluate Bone Tissue Engineered Constructs

**DOI:** 10.1155/2014/348635

**Published:** 2014-03-11

**Authors:** Livia Poser, Romano Matthys, Peter Schawalder, Simon Pearce, Mauro Alini, Stephan Zeiter

**Affiliations:** ^1^AO Research Institute Davos, Clavadelerstraß 8, 7270 Davos Platz, Switzerland; ^2^RISystem AG, Talstraße 2A, 7270 Davos, Switzerland; ^3^Vetsuisse Faculty, University of Bern, Länggassstraße 128, 3001 Bern, Switzerland

## Abstract

Tissue engineered constructs should be tested for their efficacy not only in normal but also in osteoporotic bone. The rat is an established animal model for osteoporosis and is used often for bone healing studies. In this study a defined and standardized critical size defect model in the rat suitable for screening new tissue engineered constructs in normal and osteoporotic bone is described and validated. Normal and ovariectomised Wistar rats received a unilateral middiaphyseal 5 mm defect in the femur, which was instrumented with a radiolucent PEEK plate fixed with angular stable titanium screws and left untreated. All animals were euthanized eight weeks after defect surgery and the bone healing was evaluated using radiographs, computed tomography measurements, and histology. The developed fixation system provided good stability, even in osteoporotic bone. The implants and ancillary instruments ensured consistent and facile placement of the PEEK plates. The untreated defects did not heal without intervention making the model a well-defined and standardized critical size defect model highly useful for evaluating tissue engineered solutions in normal and osteoporotic bone.

## 1. Introduction

Although bone possesses a good healing capacity, it may be limited/insufficient under certain clinical situations such as large bone defects after high energy trauma, revision surgeries, or tumour resection. Currently, bone grafting is regarded as “gold standard” to treat these cases [1–4]. However, limited availability, donor site pain, prolonged surgery time, and therefore increased risk of infection have urged researchers on developing bone substitutes [5–7]. More recently, within the concept of tissue engineering, these (osteoconductive) bone substitutes have been combined with (osteogenic) cells and/or bioactive (osteoinductive) factors. Although acute toxicity, biological activity, cytocompatibility, and fundamental biological mechanisms (e.g., transcription, translation, and signaling events and processes) can be assessed* in vitro*, such systems cannot provide a reproducible approximation of the real life* in vivo* settings. Biocompatibility, degradation properties of implant materials, survival of transplanted cells, tissue response (i.e., bone ingrowth into the construct), and mechanical function inter alia can only be investigated* in vivo.* For such investigations well-defined and standardized animal models are needed [8].

One experimental approach for the* in vivo* assessment of tissue engineered constructs is the so-called “critical size defect” (CSD) models. In the ASTM* Standard Guide for Preclinical in vivo Evaluation in Critical Sized Segmental Bone Defects* (F2721-09) CSD model is defined as “a defect that will not heal without intervention” [9]. Others define a defect as critical sized if defect does not heal spontaneously within the lifetime of the animal or experiment [10–13]. CSD models have been described for small (i.e., rat, rabbit) and large (i.e., dog, sheep, goat, and pig) animals. The latter are advantageous regarding the dimensions and biomechanical situation, but are time, labor, and cost intensive and the former are more suitable for basic research questions and screening experiments.

Bone ingrowth into the bone substitutes/tissue engineered constructs is not only dependent on the implanted construct properties, but also on the regenerative capacity of the host bone, which might be impaired by pathological conditions such as osteoporosis [14]. Since life expectancy is increasing, treatment of large bone defects in osteoporotic bone is a challenge that orthopaedic surgeons will face more often in the future. Hence, new bone substitutes/tissue engineered constructs should be tested in normal and osteoporotic bone. The rat is a well-established animal model for osteoporosis [15]. Further, 38% of articles published in six orthopaedic journals to study fracture repair in long bones (including noncritical-sized and critical sized gap defects) have used the rat as animal model [16]. Even though a number of different fixation systems for CSD in rats have been published, reproducible mechanical fixation resulting in consistent loading conditions has been difficult to achieve with these systems. Furthermore, osteoporosis leads to a reduced bone mass so that a stable fixation is even more difficult. Therefore, the goal of this study was to develop and validate* in vivo* a standardized CSD model in the rat suitable for screening new bone substitutes/tissue engineered constructs in normal and osteoporotic bone.

## 2. Materials and Methods

### 2.1. Implants and Jigs

**Figure 1:**
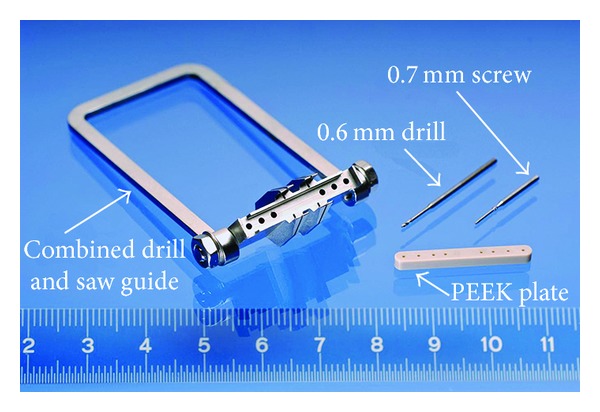
Osteosynthesis system consisting of a PEEK plate, 23 mm (length) × 2 mm (height) × 3 mm (width), which is mounted on the rat femur by six 0.7 × 5.2 mm angular stable self-tapping screws after predrilling with a 0.65 mm drill bit. To facilitate plate placement and to create a standardized 5 mm defect a combined drill and saw guide is utilized (RatFix, RiSystems).

Based on surgical anatomy, an angular stable implant system (RatFix, RISystem) with the corresponding surgical jigs (Drill- & Saw guide 5.00 mm, RISystem) was developed. The system consists of a PEEK plate, 23 mm (length) × 2 mm (height) × 3 mm (width), which is mounted on the femur by six 0.7 × 5.2 mm angular stable self-tapping screws after predrilling with a 0.65 mm drill bit using a minidrill system (AccuPen, RISystem). To facilitate plate placement and to create a standardized 5 mm defect a combined drill and saw guide is utilized (Figure 1). The defect is created with a 0.22 mm Gigli wire saw (Gigli wire saw, RISystem).

### 2.2. Experimental Design

All experiments were carried out under the licence provided by the local ethical committee (Amt für Lebensmittelsicherheit und Tiergesundheit Graubünden). Twenty- four female Wistar rats (weight, mean ± SD: 184 ± 2 g) were randomly divided into two treatment groups of twelve animals each. All animals underwent a unilateral operation to create a 5 mm middiaphyseal defect in the right femur at age of 17 weeks. All defects remained empty to serve as negative control. Group II underwent additionally ovariectomy at the age of 12 weeks to induce osteoporosis prior to defect surgery. All animals were sacrificed 8 weeks after the defect surgery at the age of 23 weeks.

### 2.3. Defect Surgery (All Animals)

Rats received 0.03 mg/kg subcutaneously (SC) buprenorphine (Essex Chemie) one hour prior to surgery and 1 mg/kg SC meloxicam (Boehringer Ingelheim) immediately before the initial skin incision. Anaesthesia was induced and maintained with 2% isoflurane (Baxter International) and a 0.3 mL/min oxygen flow. Anaesthetized rats were positioned on the operation table in lateral recumbency with the right leg facing upwards. The operation site was shaved and aseptically prepared. A lateral approach via skin incision between the greater trochanter and the knee joint was performed, and the superficial fascia was incised. The intermuscular plane between the vastus lateralis and the biceps femoris muscles was separated. The periosteum of the femur was incised. The PEEK plate was then fitted into the jig and secured using a 3-0 Vicryl (Ethicon) suture. The jig-plate assembly was subsequently fixed to the craniolateral surface of the femur by pulling the sutures through under the femur, allowing the assembly to be tightened to the femur.

After predrilling the holes using a 0.65 mm drill bit the PEEK plate was attached to the femur by six 0.7 mm angular stable bicortical titanium screws. Standardized 5 mm defects were created using a 0.22 mm Gigli wire saw guided by the sawing device of the jig. After defect sawing, the jig and bone piece were removed. The fresh defect was then flushed with sterile lactated Ringer's solution. All defects were left empty. All wounds were closed in two muscle layers with a subcutis and intracutaneous 5-0 Vicryl rapide sutures.

After surgery, rats were injected with 5 mL of warm lactated Ringer's solution intraperitoneal (IP). Every 12 hours for 3 days following surgery, rats received 0.03 mg/kg buprenorphine SC and 1 mg/kg meloxicam per os in the drinking water.

### 2.4. Ovariectomy (Group II Only)

The anaesthesia protocol used was the same as for the defect surgery, with the exception that after surgery analgesia was given for 2 days. After preparing the surgery site aseptically, a 2 cm dorsal skin incision was made halfway between the hump and the tail base. Connective tissue between the skin and the muscular layer was bluntly dissected. The ovary was grasped with a castration clamp, allowing the proximal uterine horn together with some fat to be cauterized and removed. The cavity was closed with a muscle and subcutaneous suture. The skin was closed with an intracutaneous suture. For all sutures, 5-0 Vicryl rapide (Ethicon) was used.

### 2.5. Bone Mineral Density (BMD) Evaluation

A computed tomography (CT) evaluation (XtremeCT, Scanco Medical) was performed at the level of the proximal tibial metaphysis within a distance of 2 mm distal of the patella to quantify BMD at the time of ovariectomy (T1: 0 weeks), at the time of defect creation (T2: 5 weeks), and at sacrifice (T3: 13 weeks) under isoflurane anaesthesia.In the ovariectomised group measurements took place at all three time points and in a subset of 4 rats of the nonovariectomised empty defect group (group II) at T2 and T3. Cross-sectional slices were collected with an isotropic voxel resolution of 41 *μ*m and a pixel matrix of 3072 × 3072, using an effective energy of 70 kVp and a current intensity of 900 *μ*A.

### 2.6. Radiographic Evaluation

Radiographs of the operated femurs were taken at weekly intervals after surgery under isoflurane anaesthesia. Radiographs were taken in lateromedial and craniocaudal projections to assess bone healing and to rule out implant loosening or failure.

### 2.7. Fluorochrome Injections

All rats were injected with fluorochromes at two time points to evaluate bone formation and remodelling. Rats were injected with 0.1 mg/kg calcein green (Fluka) SC 4 weeks after surgery, and with 0.1 mg/kg xylenol orange (Fluka) 7 weeks after defect surgery.

### 2.8. MicroCT Evaluation

After sacrifice CT measurements (MicroCT-40, Scanco Medical) of the defect were performed to quantify newly mineralised bone volume. The plate was adjusted parallel to the longitudinal axis of the device and scanning parameters included a source voltage of 70 kV, an intensity of 114 *μ*A, and a two-dimensional detector array with 2048 × 256 elements. The scans were performed with a resolution of 16 *μ*m.

### 2.9. Histological Evaluation

After sacrifice, femurs with attached PEEK plates were explanted and fixed in 70% ethanol. Femurs were subsequently embedded in polymethylmethacrylate (PMMA) (Fluka 64200, Fluka) and serially sectioned with a circular saw (Leitz 1600 Saw microtome,). Cross-sections of the femur parallel to the plane of the screws were ground and polished (Exact Micro Grinding System, Exakt Apparatebau). Half of the samples were stained with Giemsa Eosin (Fluka 48900/45240, Fluka) for qualitative morphological analysis, including assessment of inflammation and cell differentiation. The remaining samples were stained with Toluidine blue (Fluka 89640, Fluka) for assessment of bone, cartilage, connective tissue, and cells.

Fluorochrome labelled bone sections were assessed using a triple filter (Zeiss filter set no. 25, Zeiss Axioplan, Carl Zeiss AG) and a fluorescent lamp. The defect zones and the areas around the screws and plates were defined as the regions of interest (ROI) and systematically evaluated.

## 3. Results

### 3.1. Implants

No complications (e.g., implant failures) were observed during this study.

### 3.2. BMD Evaluation

**Figure 2:**
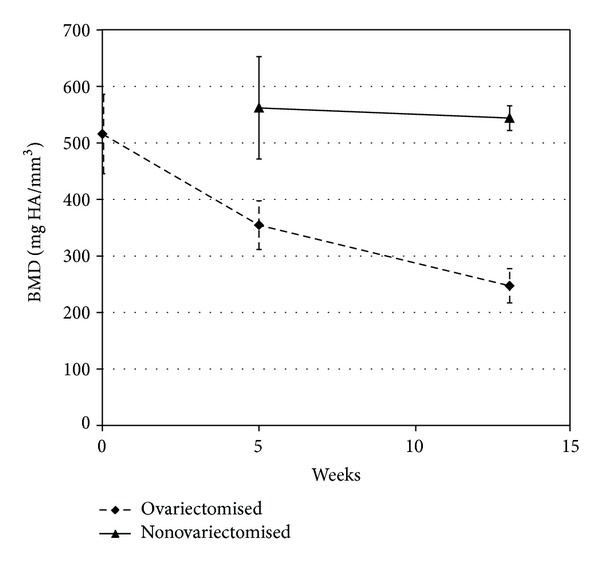
BMD loss over time (T1: 0 weeks; T2: 5 weeks; T3: 13 weeks) in the ovariectomised groups (groups II) and the nonovariectomised defect group (group I).

For the ovariectomised group II, BMD decreased from baseline values (T1: 0 weeks, 515.90 ± 70.4 mgHA/mm³) by 31% at the time of defect creation (T2: 5 weeks; 354.50 ± 43.2 mgHA/mm³) and by 52% at the time of sacrifice (T3: 13 weeks) (247.18 ± 30.2 mgHA/mm³). In contrast, in nonovariectomised defect rats BMD remained constant throughout the study: 562 ± 90.5 mgHA/mm³ at T2 and 544 ± 21.9 mgHA/mm³ at T3, respectively (Figure 2). BMD of the ovariectomised group at T2 had a *T*-score of −2.3 and at T3 of −13.6, respectively. Using the WHO definition of osteoporosis (*T*-score ≤ −2.5) the ovariectomised group was at T2 classified as osteopenic and at T3 as osteoporotic.

### 3.3. Radiographic Evaluation

**Figure 3:**
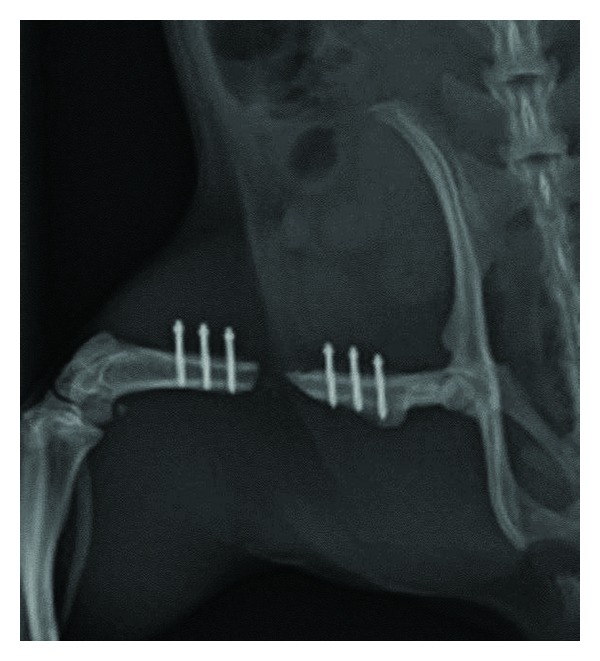
Representative radiograph of the nonovariectomised control group taken at euthanasia, 8 weeks after defect surgery.

Radiographic evaluation of the empty defect groups indicated little new bone formation until week 4 and no further progression beyond this time (Figure 3). In none of the defects bridging was observed.

### 3.4. MicroCT Evaluation

The results of the MicroCT 40 analysis quantified the radiographic findings. The volume of newly formed bone within the defect was 6.85 ± 5.51 mm³ for the nonovariectomised and 4.74 ± 3.44 mm³ for the ovariectomised group, respectively. The calculated volume of the defect size was 25 mm³ (cylinder with a diameter of 2.5 mm and a height of 5 mm).

### 3.5. Histological Evaluation

Histological analysis revealed good integration of the implant in the host tissue with no signs of screw loosening. In the nonovariectomised empty defect animals (group I) a trend towards increased bone healing compared to the ovariectomised group was observed. In the fluorochrome images, incorporation of fluorochromes demonstrated good bone formation at the defect edges and a solid integration process of the implants. This was characterized by high bone turnover, as indicated by the different regional distributions of fluorochromes around the screws.

In the fluorochrome images of the ovariectomised group (group II) little new bone formation was observed at the defect with activity around the screws being negligible. At the epiphyseal growth plate, the effects of ovariectomy were clearly visible. Both fluorochromes were barely detectable. Longitudinal growth in these animals was reduced compared to group I animals.

**Figure 4:**
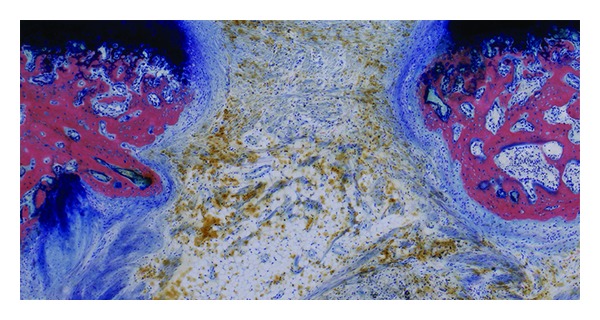
Representative image of the defect at euthanasia of the ovariectomised group. New bone formation is detected at the defect border. The defect center is filled with fat, hemosiderin, and fibrous connective tissue (Giemsa Eosin staining).

In all animals defects did not bridge and the gaps contained besides the above mentioned little bone formation mainly fat, cellular connective tissue, and some muscle tissue (Figure 4).

## 4. Discussion

Many different animal models to investigate tissue engineered constructs are used today [9]. The choice of model will mainly depend on the research question as well as on personal and institutional capabilities, experiences, and preferences. Nevertheless, there is an ethical, scientific, and economical imperative that these models are defined and standardized in order to reduce variation and hence, reduce number of animals and resources needed and to maximise validity of the obtained results. This study is not meant to exclude other models and/or methods, but rather intended to provide researchers characteristics, advantages, and limitations of this model, which might be very useful to investigate tissue engineered constructs in normal and osteoporotic bone in a standardized and well-defined fashion.

In this model, the surgical approach is straight forward and implant placement with the aid of the jig is reliable and the defect is consistently sized. The use of angular stable screws avoids compression of the underlying periosteum and has been shown to perform better in osteoporotic bone compared to conventional screws [17]. No implant associated complications were experienced in these animals, indicating that the fixation system is appropriate and safe to be used in ovariectomised rats with substantially reduced bone mass.

Another advantage of this system is the use of a biologically inert PEEK plate. During the observation period, bone defect healing can only be evaluated by X-rays. Radiopaque materials such as stainless steel or titanium do not allow for a proper visualization of the defect. Therefore, a plate made of radiolucent material is beneficial for good assessment of defect healing in all imaging planes. Furthermore, longitudinal CT measurements of the defect could be done avoiding the need to euthanize animals at different time points and hence, reducing the number of animals needed.

In this study, a 5 mm defect in the rat femur was of critical size in normal as well as in osteoporotic bone, since it did not heal without intervention. Since mainly fat, hemosiderin, and fibrous connective tissue have been observed within the defect at euthanasia, the osteogenic potential of the tissue engineered constructs being tested can be considered unequivocal. Even though 5 mm is the typical size for a diaphyseal CSD in the rat [9], it has to be emphasized that the size of a CSD depends on the rat strain, weight, age, sex, metabolism status, and the fixation system used [18]. If one of these factors is changed, the size of the defect might need to be changed to remain of critical size.

Tissue engineered constructs should enhance bone healing and ideally lead to bony union or comparable healing with the current gold standard bone graft. One limitation of the rat model is that bone graft needs to be harvested from a donor animal. Corticocancellous bone grafts from the iliac wing of donor animals lead in some animals to healing of the defect, but in most cases sequestering and resorption of the allograft with giant cells were observed (data not shown). Wistar rats are an outbreed stock so that most likely genetic homology was insufficient to allow for allogenic bone grafting. Inbreed strain rats such as Fischer 344 are considered syngenic and successful bone grafting from donor rats to treat large bone defect has been described [19]. Another possibility would be the use of athymic (“nude”) rats lacking T cell mediated immune response for studies in which a positive control group is required or cells of human origin are used.

## 5. Conclusion

This well-defined and standardized system will be highly useful for tissue engineered solutions for the treatment of large bone defects in normal and osteoporotic bone.
